# Intravenous Lidocaine Compared to Fentanyl in Renal Colic Pain Management; a Randomized Clinical Trial

**DOI:** 10.22037/emergency.v5i1.18894

**Published:** 2017-10-23

**Authors:** Hassan Motamed, Mohammadreza Maleki Verki

**Affiliations:** 1 Emergency Medicine Department, Ahvaz Jundishapur University of Medical Sciences, Ahvaz, Iran.

**Keywords:** Fentanyl, lidocaine, renal colic, pain management, analgesia, emergency service, hospital

## Abstract

**Introduction:**

Using alpha blockers such as intravenous (IV) lidocaine has been deemed effective in controlling acute pain. Therefore, the current study was designed with the aim of evaluating the efficiency of IV lidocaine in comparison to IV fentanyl in pain management of patients with renal colic in emergency department (ED).

**Methods:**

In this double blind clinical trial, 18-65 year old patients that presented to ED with colicky flank pain and met the inclusion criteria of the study were allocated to either lidocaine or fentanyl group using block randomization and compared regarding pain severity 5, 10, 15, and 30 minutes after drug administration.

**Results:**

90 patients with the mean age of 35.75±8.87 years were divided into 2 groups of 45 (90% male). The 2 groups were not significantly different regarding the studied baseline variables. Pain severity was not significantly different between the 2 groups at various times after injection. Treatment failure rate 15 minutes after injection was 44.4% (20 cases) in IV lidocaine and 17.8% (8 cases) in IV fentanyl group (p = 0.006). These rates were 26.6% (12 patients) versus 22.2% 30 minutes after injection (p = 0.624). Absolute risk increase of treatment failure in case of using lidocaine was 26.7 (95% CI: 8.3-44.9) in the 15^th^ minute and 4.4 (95% CI: 13.3-22.2) 30 minutes after injection. Number needed to harm (NNH) in treatment with lidocaine 15 and 30 minutes after injection were 4 (95% CI: 2.2-12.0) and 23, respectively.

**Conclusion:**

Although mean pain severity was not significantly different between IV fentanyl and lidocaine at various times after injection, treatment failure rate was significantly higher in the IV lidocaine group 15 minutes after injection.

## Introduction

Renal colic is one of the most common clinical manifestations of a stone being present in the urinary system, which presents as sudden and severe flank pain (1). In the United States, the prevalence of renal colic has increased from 5.2% during 1994-1998 to 8.8% in 2007-2010 (2, 3). 

One of the major duties of emergency department (ED) is reducing patients’ pain and suffering before taking any treatment or surgical measures. 

Recently, using alpha blockers such as intravenous (IV) lidocaine, nifedipine and nerve blockers in the intercostal area has been deemed effective in reducing renal colic pain (4-6). 

When narcotic drugs lack the required effectiveness and lead to undesirable side effects, lidocaine can be a good choice. IV lidocaine has been deemed effective in controlling neuropathic pains such as diabetic neuropathy, post-surgery pains, bone fracture pain, headache and nervous system malignancies (7-10). Continuous infusion of IV lidocaine during and after abdominal surgery has accelerated patient recovery and reduced length of hospital stay (11). 

Using opioids has some dangers due to reasons such as inhibition of respiratory center in medulla region and activation of vomiting center (12). These drugs are used as an appropriate analgesic in ED either alone or along with midazolam (13). 

Finding an effective analgesic with minimal side effects has been continuously desired by the physicians involved with these patients. Therefore, the current study was designed with the aim of evaluating the efficiency of IV lidocaine in comparison to IV fentanyl in pain management of patients with renal colic.

## Methods

***Study design and setting***

In this double blind clinical trial, the effectiveness of IV lidocaine and IV fentanyl was evaluated and compared in pain management of patients with renal colic admitted to ED of Golestan Hospital, Ahwaz, Iran, in 2015. The study was approved by the ethics committee of Ahwaz University of Medical Sciences under the number “ajums.REC.1392.324” and the researchers adhered to all the principles stated in the declaration of Helsinki regarding ethical practice and confidentiality of patient data. Informed consent was obtained from all the participants for taking part in the study. All the expenses of patients’ treatment were covered by the project executive and no additional fees were inflicted upon the patients. This study was registered on the Iranian registry of clinical trials under the number IRCT2017081415446N12.

***Participants***

Patients in the 18-65 years age range that had presented to the ED with colicky flank pain and lacked histories such as: cardiac dysrhythmia and ischemia, parenchymal tissue problems in liver and kidney, and history of using mono amino oxidase (MAO) inhibitor drugs in the last 2 weeks, were included in the study. In addition, patients with a history of allergy to morphine or other opiates, definite or possible pregnancy, lactating women, addiction to opiates, and receiving analgesics in the last 6 hours were excluded from the study. To confirm absence of dysrhythmia or underlying ischemic disease, electrocardiogram was used on admission. All the clinical examinations were done by 2 physicians, one senior resident of emergency medicine and one senior resident of urology.

Clinical diagnoses were confirmed by performing ultrasonography or spiral computed tomography (CT) scan, or presence of hematuria in urinalysis after management of the patient’s pain and those who did not have definitive evidence of stone in evaluations were excluded from the study.

***Intervention***

Patients were allocated to a group receiving either lidocaine (1.5 mg/kg) or fentanyl (1.5 µg/kg) via block randomization. Drug prescriptions were as IV infusion during 2 minutes while patients were under cardiac monitoring. For patients who still had moderate to severe pain 30 minutes after injection, morphine sulfate with the standard dose of 0.1 mg/kg was prescribed as additional analgesic. The physician prescribing the drug and the patient were blind to the prescribed drug. Drugs were prepared by a nurse in syringes with the same volume and color in the absence of the physician and were then given to the physician.

***Outcome***

The primary outcome of this study was evaluating the pain score of patients based on visual analog pain scale (VAS) 5, 10, 15, and 30 minutes after injection. 3 points pain reduction based on VAS was considered as clinically significant pain reduction. Therefore, lack of 3 points pain reduction 15 and 30 minutes after injection were considered as treatment failure.

***Data gathering***

Demographic data (age, sex, weight) and data regarding pain severity on admission to ED and 5, 10, 15, and 30 minutes after injection were gathered using a checklist. The senior emergency medicine resident was responsible for data gathering and was blind to the drug received by the patient.

***Statistical analysis***

Sample size was estimated to be 40 for each group considering 95% confidence interval and type 2 error of 0.2% (4). Data analysis was done using SPSS 21 software. Quantitative data were reported based on mean ± standard deviation and qualitative ones based on frequency and percentage. Chi square test, Fisher’s exact test and t-test were used for comparisons. P values less than 0.05 were considered significant.

## Results

***Baseline characteristics***

90 patients with the mean age of 35.75±8.87 years (20-55) were randomly divided into 2 groups of IV lidocaine (45 patients) and IV fentanyl (45 patients) (90% male). Table 1 has compared the baseline characteristics of the 2 groups. As can be seen, the 2 groups are not significantly different regarding studied baseline variables. 

***Pain management***

**Figure 1:**
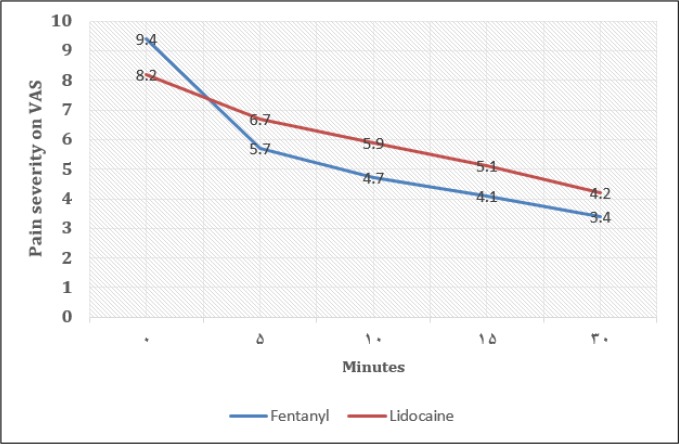
Comparison of pain severity between the 2 studied groups 5 (p = 0.113), 10 (p = 0.056), 15 (p = 0.137) and 30 (p = 0.291) minutes after drug injection.

Table 2 and figure 1 compare pain severity between the 2 groups 5, 10, 15, and 30 minutes after drug injection. Pain severity was not significantly different between the 2 groups at various times after injection. 

Treatment failure rate 15 minutes after injection was 44.4% (20 cases) in IV lidocaine and 17.8% (8 cases) in IV fentanyl group (p = 0.006). These rates were 26.6% (12 cases) versus 22.2% (10 cases), 30 minutes after injection (p = 0.624). 

Therefore, the absolute risk increase of treatment failure in case of using lidocaine was 26.7 (95% CI: 8.3-44.9) in the 15^th^ minute and 4.4 (95% CI: 13.3-22.2) 30 minutes after injection. 

Number needed to harm (NNH) in treatment with lidocaine 15 and 30 minutes after injection were 4 (95% CI: 2.2-12.0) and 23, respectively.

## Discussion

Based on the findings of the present study, although mean pain severity was not significantly different between IV fentanyl and IV lidocaine groups at various times after injection, treatment failure rate was significantly higher in the lidocaine group 15 minutes after injection.

**Table 1:** Comparison of baseline characteristics between the 2 studied groups

**Variable **	**IV fentanyl**	**IV lidocaine**	**P value**
**Sex **			
Male	39 (86.7)	42 (93.3)	0.292
Female	6 (13.3)	3 (6.7)	
**Age (year)**	39.08 ± 6.64	34.08 ± 9.49	0.112
**Weight (kg)**	80.93 ± 15.27	82.85 ± 15.83	0.572
**Pain severity on admission**			
Moderate	2 (4.4)	2 (4.4)	1.000
Severe	43 (95.6)	43 (95.6)	

Data are presented as frequency (%) or mean ± standard deviation. IV: intravenous.

**Table 2:** Comparison of pain severity between the 2 studied groups at various times after drug injection

**Time **	**IV fentanyl**	**IV lidocaine**	**P value**
**5 minutes**			
Mild	8 (17.8)	7 (15.6)	0.302
Moderate	17 (37.8)	11 (24.4)	
Severe	20 (44.4)	27 (60.0)	
**10 minutes**			
Mild	14 (31.1)	11 (24.4)	0.310
Moderate	18 (40.0)	14 (31.1)	
Severe	13 (28.9)	20 (44.4)	
**15 minutes**			
Mild	20 (44.4)	14 (31.1)	0.405
Moderate	14 (31.1)	16 (35.6)	
Severe	11 (24.4)	15 (33.3)	
**30 minutes**			
Mild	25 (55.6)	22 (48.9)	0.679
Moderate	7 (15.6)	10 (22.2)	
Severe	13 (28.9)	13 (28.9)	

Data are presented as frequency (%). IV: intravenous.

In addition, the absolute risk increase of treatment failure in case of using lidocaine 15 and 30 minutes after injection were 26.7 and 4.4 percent, respectively.

Renal colic is caused due to increased pressure in the upper urinary tract or dilatation of kidney capsule following urinary retention. 

In a study by Khaniha et al. on evaluating the effect of various drugs in relieving renal colic, the results showed that pethidine 10 to 45 minutes after injection, methadone 30-60 minutes and morphine 1.5 to 30 minutes after injection showed their analgesic effects (14). 

Using intranasal fentanyl led to an effective sedation in patients 30 minutes after administration (15). In another study that had compared the effectiveness of lidocaine and morphine, the findings showed that lidocaine is a safe, effective and cheap method for induction of analgesia in patients with renal colic compared to morphine, which lacks the side effects of morphine such as nausea and vomiting. Time needed for induction of analgesia when using morphine alone and morphine with lidocaine were reported to be 100 and 87 minutes after injection, respectively (16). In another study, 240 patients aged 18 to 65 years presenting to Imam Reza Hospital, Tabriz, Iran, with renal colic were randomly divided into 2 groups receiving either IV morphine or IV lidocaine. The results indicated effectiveness of IV lidocaine in comparison to morphine (4).

Based on the results of this study, it seems that IV lidocaine has proper ability in controlling renal colic during 30 minutes. However, if the speed of analgesia induction is of higher priority for the physician and patient compared to probable side effects, considering the high rate of treatment failure of IV lidocaine in 15 minutes (44.4% vs 17.8% for fentanyl), it cannot be a good choice for this purpose.

## Conclusion

The absolute risk increase of renal colic management failure with IV lidocaine 15 and 30 minutes after injection were 26.7 and 4.4, respectively. It seems that IV lidocaine cannot be a good choice when quick pain control is of higher priority for the physician.

## Limitations

Stone size, history of kidney stone, and ethnic characteristics were not evaluated in this study, but they may affect pain severity and response to analgesics used.
